# Supplementary material for: Chromothripsis during telomere crisis is independent of NHEJ, and consistent with a replicative origin
Source: Genome Res. 2019 May;29(5):737–49. doi: 10.1101/gr.240705.118 (PMC6499312; doi:10.1101/gr.240705.118)
Supplement: Supplemental Material [file supp_gr.240705.118_Supplemental_file_1.zip › contigs/annotated_contigs/DB49/contig.2.DB49_length_797_mean_cov_12.0225846926.docx]

**DB49_length_797_mean_cov_12.0225846926**

```
GCAGGAGATCTTTGGGTCAGTGGATTCAAGAAGGTAATTATTCACTGAGGTGCATTTCGTACACTTGGTCATGCCTTACCCTTGGGCAT
 >chr6:125988798-125989136 - E=2e-185
TGTATGTGTTGAATCATTTTCTTTTTATTAGCCATGAAAATAATCACTCTGTGTTGGGCTGTCATTGTGTTCTATTTTTCTGGGATTTT

AATTTTTTAAAAATTGCCTTTCGCTAACTTGAAAACAATGTGTTTTCAGGCTCCAGGTTGTCTGCTCACTAGTGAGTGGGGTCTTGGGT

CAGGTCATTCAGATTCCCACAGGATTCATTACTCACCTACATTGTGGTCTGCCAATGCACACCATAT|ACCA|TTGAAACAAGTCAGTA
                                                                    >chr6:126298589-126
ACAAAATATTCACATAACTGTATCACAGATCTTAGGAAACAGACATTCAGAAAAGATTTAAGGCCACTAAGTAACAGCCTCTCATAAAA
299027 - E=1e-236
CCCAACAAATCTTAAAATTGCAATTAGACTTAAAAGGGACCTAAATACCACTTCATGCTGAACCAAGATTAGAAAAATCTTCCACTCTT

GACATTTTCATGTTCTTAGTTTTTCAATCAAGTGATCAGCCTGTAATTAAAAAACATACTTTTTAATTCTAGATATCATAGTCTCTATC

AAATAATTCTACTGGTATCCCTATTATAATATGCCACATTAGAAGAGTTGAGTACTATATATTCTGTTCCTAAAAATATCTTACAAAAA

AACTCAGTATAATATCTATATATAAATATTTTACATTGTATATAAATAAGCAGATTTTTGCC|AACAATTCAAATTCAATACTAAGTA
```
